# Multiple Sclerosis Patients in Saudi Arabia: Prevalence of Depression and its Extent of Severity

**DOI:** 10.7759/cureus.7005

**Published:** 2020-02-15

**Authors:** Hamad Alhussain, Abdulaziz A Aldayel, Abdulaziz Alenazi, Faris Alowain

**Affiliations:** 1 Medicine, Imam Mohammad Ibn Saud Islamic University, Riyadh, SAU

**Keywords:** multiple sclerosis, severity, depression, saudi arabia

## Abstract

Background

Multiple sclerosis (MS) is a serious chronic autoimmune disorder of the central nervous system of unknown etiology. MS-related depression is a common mood disorder recognized within the medical community. However, their association is ambiguous, underdiagnosed, undertreated and less reported.

Objectives

The study aimed to estimate the point prevalence and severity of depression among multiple sclerosis patients in Saudi Arabia.

Materials and methods

We conducted an observational cross-sectional study among multiple sclerosis patients in Riyadh region, Saudi Arabia. Patients filled demographic data and Patient Health Questionnaire-9 (PHQ-9) to determine depression. Those who did not meet the age, disease duration, and regular follow-up eligibility criteria were excluded from the study.

Results

We enrolled 238 MS patients in the study, male patients represented 39.1% (n = 93) while females accounted for 60.9% (n = 145) (male:female ratio 0.64). The mean age of the study population was 32.07 ± 7.93 years. The mean duration of the disease was 7.06 ± 4.7 years. We determined that 89.9% (n = 214) of the patients showed mild to severe depression symptoms (55.46% of the females, and 34.4% of the males; p = 0.474). We further found that 37.39% (n = 89) and 65.13% (n = 155) of the depressed patients were unemployed (p = 0.039) and were non-smokers (p = 0.097) respectively. Furthermore, depression severity is significantly associated with education (p = 0.005).

Conclusion

High levels of depression symptoms were found among MS patients in Saudi Arabia. The relationship between MS and psychiatric conditions exists despite the uncertainty of its pathogenesis. Further longitudinal studies should be carried out to obtain more valid outcomes. Neurologists treating MS patients can play a role in studies related to the condition by investigating depressive symptoms actively and providing the data.

## Introduction

Over the last decades, the global interest in the burden of different chronic diseases and depression and the relationship between them has been increasing dramatically [1,2]. Multiple sclerosis (MS), one of the chronic disorders, is an autoimmune disorder of the central nervous system (CNS) of unknown etiology. The condition is characterized by demyelination and variable degrees of axonal loss that leads to significant neurological disability [3,4]. The pathogenesis of MS involves immune response directed against CNS antigens mediated through activated CD4+ myelin-reactive T cells and the possible involvement of B cells [3]. This immunopathogenesis possibly has a persistent peripheral activation of autoreactive T cells from the breakdown of self-tolerance of myelin and other CNS antigens [5]. Self-tolerance breach can be triggered in an individual with genetic susceptibility due to low vitamin D levels and environmental antigens, presumptively infection with Epstein Barr virus (EBV) [3,5]. Also, systematic reviews and meta-analysis studies comprehensively established the association between smoking habits and dosage of smoking as potential risk factors for MS development and progression [6,7]. MS is considered as one of the most common neurological disorders and causes of disability in young adults [8]. Globally, the prevalence of MS in 2008 was 2.1 million with a median of 30 cases per 100,000. The figure increased to 2.3 million with a median of 33 cases per 100,000 in 2013 [8]. The prevalence of MS differs with geographical variation and ethnicity. The median prevalence of MS per 100,000 is 80 in Europe, 14.9 in Eastern Mediterranean, 8.3 in the Americas, 5 in Western Pacific, 2.8 in South-East Asia, and 0.3 in Africa. The median estimated prevalence of MS in Saudi Arabia is 20.01 to 60 people per 100,000 [8]. The global average age of onset of MS symptoms is 29.2 years, with an interquartile range between 25.3 and 31.8 years, while the global male:female ratio of MS is 0.5 (two women for every single man) [8]. The concept of depression has developed from lowness of spirits of persons suffering under disease to major depressive disorder (MDD) or depression within the framework of mood disorders [9,10]. MDD is characterized by a depressed mood and diminished interest or pleasure in almost all activities throughout the day for at least two weeks [9]. The pathophysiology of depression remains uncertain but some researchers hypothesized several theories to explain the depression mechanism. It is believed that low levels of brain-derived neurotrophic factor (BDNF) leads to depression according to the neurotrophin hypothesis, but evidence of the relationship is still debatable [11]. The monoamines hypothesis provides stronger evidence by showing that the underlying pathophysiological origin of depression is due to low concentrations and depletion of serotonin, norepinephrine, and dopamine in the CNS [12]. The pathogenesis of depression in MS remains vague. Regarding epidemiology, prevalence, age of onset, and the male:female ratio of depression, they differ across the world populations [13]. The global lifetime prevalence in 2015 was estimated around 322 million or 4.4% of the world population (World Health Organization, WHO), which was 18.4% more compared to the 2005 statistics [1]. Depression manifests more in females compared to males (male:female ratio 1.6) (WHO) [13]. Depression has two peaks for age of onset, the first peak is in older adulthood ranging from 55 to 74 years (WHO), with diversity in determining the second peak as in childhood and adolescents below the age of 15 years (WHO) and to be in late 40s for men and middle 30s for women [14]. The evidence of MDD epidemiology in Saudi Arabia is lacking but the estimated prevalence is consistent with that of the Middle East and Africa at 6.6% [15]. MDD is the third commonest health problem that causes disability in Saudi Arabia [2]. In 2018, depression was named the leading cause of world disability (WHO) and is expected to be the major contributor to disease burden by 2030 (WHO, 2008) [16]. Depression has an influence on the general health and quality of life (QOL) [2]. Risk of suicide differs significantly among depressed and normal individuals, between 2% to 7% of depressed adults are having risk to commit suicide. While normal population range is estimated at 0.5% to 1.4% or approximately 12 per 100,000 individuals per year. Several studies have attempted to address the correlation between MS and depression. In Norway, the prevalence of depression symptoms among MS patients is significantly higher than the general population at 31.4%. Accordingly, 16% of the depressed untreated patients articulated the need for antidepressant treatment [17]. Another study that aimed to determine the lifetime risk of depression in a representative group of MS patients in North America reported similar results. The study concluded that 34.4% had a current or lifetime diagnosis of depression [18]. Moreover, other studies have been done to estimate the prevalence of depression symptoms in MS patients in Tunisia and Iran and they reported a prevalence of 65% and 59.4%, respectively [19,20]. There is limited current literature about the relationship between depression and multiple sclerosis in the Saudi community. This study is meant to address the point prevalence of depression and its severity among multiple sclerosis patients in Saudi Arabia.

## Materials and methods

Study design and subjects

This is an observational cross-sectional study conducted to assess depression point prevalence and its severity among multiple sclerosis patients in Saudi Arabia. The recruited sample is Saudi MS patients in Riyadh region, Saudi Arabia. Snowball sampling as designated by Goodman was used to recruit the participants online [21]. Those who have depression preceding the diagnosis of MS, with less than one year of MS symptomatology, aged less than 15 years and older than 50 years, and have no regular follow-up with a neurologist were excluded from the study.

Data collection tool and research instrument

Data collection was done through a self-administered anonymous online questionnaire that was divided into two parts. The first section concentrated on socio-demographic data of the participants, including age, gender, education level, marital status, and disease duration. The second part aimed to measure the point prevalence and severity of depression using the Patient Health Questionnaire-9 (PHQ-9). We used the standardized, valid, and reliable Arabic version of the instrument for depression screening and diagnosis consisting of nine statements based on DSM-IV [22]. The statements are set as 4-point Likert scale items and graded from 0 to 3 (not at all, several days, more than half the days, and nearly every day) to determine behavior in the past two weeks. Each application was organized and given a score between 1 to 27 as follows: 0-4 no or minimal depression, 5-9 mild depression, 10-14 moderate depression, 15-19 moderately severe depression and ≥20 is severe depression [22,23]. An additional conclusive statement is included at the end of the diagnostic tool as a severity measure asserting, “How difficult have these problems made it for you to do your work, take care of things at home, or get along with other people?” The expected answers were not difficult at all, somewhat difficult, very difficult and extremely difficult [23].

Data analysis

Data were coded and entered into the computer, Microsoft Excel Software and analyzed using SPSS version 25.0 (IBM Corp., Armonk, NY). Descriptive statistics were expressed as counts (percentage) for categorical data while mean ± SD was used for the count data. Chi-Square test of independence was used to determine P-values of the association between the presence of depression and sociodemographic factors as well as the association between depression severity and sociodemographic factors.

Ethical approval

All participants were informed of the objectives of the study and they participated voluntarily. A bilingual informed consent in the local language (Arabic) and English was embedded in the online data collection tool. Institutional Review Board (IRB) approval was obtained to ensure the research was conducted according to the Declaration of Helsinki. Participant anonymity was guaranteed by assigning each respondent a code number for analysis.

## Results

Sociodemographic data

After application of the inclusion and exclusion criteria, a total of 238 Saudi MS patients were enrolled in the current study. The male population represented 39.1% (n = 93) while the female one was 60.9% (n = 145) (male:female ratio was 0.64). The mean age of the study population was 32.07 ± 7.93 years while the mean duration of the disease was 7.06 ± 4.7 years. Most of the participants were inhabitants of urban areas (81.5%), had a bachelor’s degree (61.8%), employed (42.4%) and never smoked a cigarette (74.4%). The average household income was almost equally dispersed for each stratum as most respondents earned less than 5000 Saudi Riyal (22.7%). Other sociodemographic characteristics are detailed in Table 1.

**Table 1:** Demographic data of the participants

Sociodemographic characteristics	n (%)
Gender	
Male	93 (39.1)
Female	145 (60.9)
Age, years (± SD; median)	32.07 ± 7.93; 32.0
Marital Status	
Single	111 (46.6)
Married	107 (45)
Divorced	17 (7.1)
Widowed	3 (1.3)
Residency	
Urban	194 (81.5)
Rural	44 (18.5)
Education	
Primary school	2 (0.8)
Intermediate school	6 (2.5)
Secondary school	61 (25.6)
University or college	147 (61.8)
Postgraduate	22 (9.2)
Household Income	
Less than 5000 Saudi Riyal	54 (22.7)
5000 - 10000 Saudi Riyal	47 (19.7)
10001 - 15000 Saudi Riyal	52 (21.8)
15001 - 20000 Saudi Riyal	35 (14.7)
20001 - 25000 Saudi Riyal	16 (6.7)
More than 25000 Saudi Riyal	34 (14.3)
Employment Status	
Employed	101 (42.4)
Unemployed/housewife	95 (39.9)
Student	42 (17.6)
Duration of the Disease, years (± SD); median	7.06 ± 4.7; 7.0
Smoking Status	
Heavy smoker	37 (15.5)
Social smoker	24 (10.1)
Not a smoker	177 (74.4)

Depression scores

Based on the total score > 4 cut-off point of PHQ-9, a total of 89.9% (n = 214) of the patients showed depression symptoms albeit varying from mild to severe stages (55.46% of the females, and 34.4% of the males; p = 0.474), while the remaining 10.1% (n = 24) did not show depression symptoms. Prevalence of different stages of depression is evenly distributed but the most common was mild (score 5-9) representing 24.8% followed by moderate 23.9%, moderately severe 22.3%, and severe 18.9%. In terms of sociodemographic factors associated with depression after using Chi-Square test of independence, a total of 37.39% (n = 89) of the depressed patients were unemployed (p = 0.039) and 65.13% (n = 155) of the depressed patients were not smokers (p = 0.097). Furthermore, depression severity is significantly associated with education (p = 0.005). Additional information about the presence of depression, its scores, and relationship with sociodemographic factors are specified in Tables 2-5.

**Table 2:** Presence of depression

Presence of depression symptoms	n (%)
No	24 (10.1)
Yes	214 (89.9)
Total	238 (100)

**Table 3:** Frequency distribution of depression severity

Depression severity	n (%)
None-minimal depression	24 (10.1)
Mild depression	59 (24.8)
Moderate depression	57 (23.9)
Moderately severe depression	53 (22.3)
Severe depression	45 (18.9)
Total	238 (100)

**Table 4:** Presence of depression symptoms associated with sociodemographic factors Note: Based on Chi-Square test of independence.

Sociodemographic characteristics	Presence of depression symptoms		P-Value
	No	Yes	
Gender			
Male	11 (4.622)	82 (34.45)	0.474
Female	13 (5.46)	132 (55.46)	
Marital Status			
Single	6 (2.52)	105 (44.12)	0.118
Married	16 (6.72)	91 (38.24)	
Divorced	2 (0.84)	15 (6.30)	
Widowed	0 (0)	3 (1.26)	
Residency			
Urban	21 (8.82)	173 (72.69)	0.426
Rural	3 (1.26)	41 (17.23)	
Education			
Primary school	0 (0)	2 (0.84)	0.107
Intermediate school	1 (0.42)	5 (2.10)	
Secondary School	2 (0.84)	59 (24.79)	
University or college	16 (6.72)	131 (55.04)	
Postgraduate	5 (2.10)	17 (7.14)	
Household Income			
Less than 5000 Saudi Riyal	4 (1.68)	50 (21.01)	0.959
5000 - 10000 Saudi Riyal	6 (2.52)	41 (17.23)	
10001 - 15000 Saudi Riyal	5 (2.10)	47 (19.75)	
15001 - 20000 Saudi Riyal	4 (1.68)	31 (13.03)	
20001 - 25000 Saudi Riyal	2 (0.84)	14 (5.88)	
More than 25000 Saudi Riyal	3 (1.26)	31 (13.03)	
Employment Status			
Employed	16 (6.72)	85 (35.71)	0.039
Unemployed/housewife	6 (2.52)	89 (37.39)	
Student	2 (0.84)	40 (16.81)	
Smoking Status			
Heavy smoker	2 (0.84)	35 (14.71)	0.097
Social smoker	0 (0)	24 (10.08)	
Not a smoker	22 (9.24)	155 (65.13)	

**Table 5:** Depression severity associated with sociodemographic factors Note: Based on Chi-Square test of independence.

Sociodemographic characteristics	Depression severity					P-Value
	None-minimal	Mild	Moderate	Moderately severe	Severe	
Gender						
Male	11 (4.62)	22 (9.24)	22 (9.24)	18 (7.56)	20 (8.4)	0.796
Female	13 (5.46)	37 (15.55)	35 (14.71)	35 (14.71)	25 (10.5)	
Marital Status						
Single	6 (2.52)	28 (11.76)	27 (11.34)	32 (13.45)	18 (7.56)	0.326
Married	16 (6.72)	28 (11.76)	24 (10.08)	17 (7.14)	22 (9.24)	
Divorced	2 (0.84)	2 (0.84)	5 (2.1)	3 (1.26)	5 (2.1)	
Widowed	0 (0)	1 (0.42)	1 (0.42)	1 (0.42)	0 (0)	
Residency						
Urban	21 (8.82)	51 (21.43)	46 (19.33)	40 (16.81)	36 (15.13)	0.575
Rural	3 (1.26)	8 (3.36)	11 (4.62)	13 (5.46)	9 (3.78)	
Education						
Primary school	0 (0)	0 (0)	1 (0.42)	0 (0)	1 (0.42)	0.005
Intermediate school	1 (0.42)	0 (0)	0 (0)	0 (0)	5 (2.1)	
Secondary School	2 (0.84)	15 (6.3)	20 (8.4)	14 (5.88)	10 (4.2)	
University or college	16 (6.72)	40 (16.81)	29 (12.18)	37 (15.55)	25 (10.5)	
Postgraduate	5 (2.1)	4 (1.68)	7 (2.94)	2 (0.84)	4 (1.68)	
Household Income						
Less than 5000 Saudi Riyal	4 (1.68)	11 (4.62)	12 (5.04)	18 (7.56)	9 (3.78)	0.427
5000 - 10000 Saudi Riyal	6 (2.52)	11 (4.62)	8 (3.36)	13 (5.46)	9 (3.78)	
10001 - 15000 Saudi Riyal	5 (2.1)	15 (6.3)	12 (5.04)	7 (2.94)	13 (5.46)	
15001 - 20000 Saudi Riyal	4 (1.68)	9 (3.78)	14 (5.88)	6 (2.52)	2 (0.84)	
20001 - 25000 Saudi Riyal	2 (0.84)	3 (1.26)	2 (0.84)	4 (1.68)	5 (2.1)	
More than 25000 Saudi Riyal	3 (1.26)	10 (4.2)	9 (3.78)	5 (2.1)	7 (2.94)	
Employment Status						
Employed	16 (6.72)	29 (12.18)	20 (8.4)	18 (7.56)	18 (7.56)	0.133
Unemployed/housewife	6 (2.52)	22 (9.24)	28 (11.76)	23 (9.66)	16 (6.72)	
Student	2 (0.84)	8 (3.36)	9 (3.78)	12 (5.04)	11 (4.62)	
Smoking Status						
Heavy smoker	2 (0.84)	6 (2.52)	5 (2.1)	14 (5.88)	10 (4.2)	0.031
Social smoker	0 (0)	10 (4.2)	5 (2.1)	4 (1.68)	5 (2.1)	
Not a smoker	22 (9.24)	43 (18.07)	47 (19.75)	35 (14.71)	30 (12.61)	

## Discussion

The medical care has a lot to be reviewed and improved within the framework of the association between chronic diseases and psychiatric disorders such as depression. The development of psychiatric conditions is still an area of research and remains unknown, giving the opportunity for several interpretations to rise. Some early studies have shown that radiologically hyperintense lesions in prefrontal and temporal cortical regions lead to structural changes responsible for depression among MS patients [24]. Others have found that lesions in cortical and subcortical projections are responsible for the pathogenesis of depression [25]. We aimed to assess the point prevalence of depression and its severity within a sample of MS patients in Saudi Arabia. Our approach differs from others in the literature since it operates a modified screening tool dedicated to the illness severity. In addition, the prevalence of depression in our model of MS patients was high probably because of maltreatment or failure of pharmacological and non-pharmacological means during the management plan. Our study findings indicated that the point prevalence of depression in MS patients was (89.9%), which is higher than in Norway (31.4%), Canada (34.4%), the Islamic Republic of Iran (59.4%), as well as the reported number of the general population in the geographical region (6.6%) [15,17,18,20]. In the current study, high frequency of MS patients with mild-moderate depression was found, which is consistent with the results of the previous studies [20,26]. Moreover, the frequency of severe level of depression in our study was 18.9%. Similar results were also found in Iran where they reported that 18.1% of MS patients had severe depression [20]. The little variation could be attributed to the demographic and environmental similarities between the two countries. On the other hand, the prevalence of severe depression is low when compared to Tunisian MS patients which was 25% [19]. Our results showed differences in terms of gender discrepancy since the female MS patients reported more vulnerability to manifest depression symptoms compared to males. The findings are compatible with the Iranian study and marginally consistent with the Norway study [17,20]. However, our data does not have a statistically supported assumption despite the unique sociodemographic morphology and gender role the Saudi women play when compared to Western communities. We noted that there were no variations in the frequency of reporting depressive symptoms regardless of the current state of marital status. Similarly, patients who are currently unemployed or housewives had a higher frequency of reporting depressive symptoms compared to students or employed patients. Furthermore, there were significant differences in the frequency of reporting depressive symptoms and patients’ smoking status. However, our findings are inconsistent with recent publications [27,28]. Lastly, our results show that higher level of education had a positive correlation with depression severities, which contradicts existing literature [20].

Study limitations

The results of the present study need to be evaluated carefully since we did not address the issue of social support and side effects of MS medications. Moreover, the cross-sectional nature of our data does not establish cause and effect relationship between the variables. The study was quasi population-based with a relatively small sample. In addition, PHQ-9 does not screen for other psychiatric comorbidities that may have a similar impact such as anxiety.

## Conclusions

High levels of depression symptoms were recorded among MS patients in Saudi Arabia. The relationship between MS and psychiatric conditions does exist despite the uncertainty of its pathogenesis. There is a need for further longitudinal studies and clinical trials to validate the impact of depression on MS patients in Saudi Arabia. The MS treating neurologists should actively investigate depressive symptoms at the time of diagnosis and throughout the disease management course to provide vital data.
